# Notch Signaling Regulates Mitochondrial Metabolism and NF-κB Activity in Triple-Negative Breast Cancer Cells via IKKα-Dependent Non-canonical Pathways

**DOI:** 10.3389/fonc.2018.00575

**Published:** 2018-12-04

**Authors:** Fokhrul Hossain, Claudia Sorrentino, Deniz A. Ucar, Yin Peng, Margarite Matossian, Dorota Wyczechowska, Judy Crabtree, Jovanny Zabaleta, Silvana Morello, Luis Del Valle, Matthew Burow, Bridgette Collins-Burow, Antonio Pannuti, Lisa M. Minter, Todd E. Golde, Barbara A. Osborne, Lucio Miele

**Affiliations:** ^1^Louisiana State University Health Sciences Center, Stanley S. Scott Cancer Center, New Orleans, LA, United States; ^2^Department of Genetics, Louisiana State University Health Sciences Center, New Orleans, LA, United States; ^3^Department of Pharmacy, University of Salerno, Salerno, Italy; ^4^Department of Pathology, The Shenzhen University School of Medicine, Shenzhen, China; ^5^Department of Medicine, Tulane University School of Medicine, New Orleans, LA, United States; ^6^Department of Veterinary and Animal Sciences, University of Massachusetts at Amherst, Amherst, MA, United States; ^7^Department of Neuroscience, McKnight Brain Institute, University of Florida at Gainesville, Gainesville, FL, United States

**Keywords:** notch, jagged, triple-negative breast cancer, mitochondria, NF-κB, cancer stem-like cells (CSCs)

## Abstract

Triple negative breast cancer (TNBC) patients have high risk of recurrence and metastasis, and current treatment options remain limited. Cancer stem-like cells (CSCs) have been linked to cancer initiation, progression and chemotherapy resistance. Notch signaling is a key pathway regulating TNBC CSC survival. Treatment of TNBC with PI3K or mTORC1/2 inhibitors results in drug-resistant, Notch-dependent CSC. However, downstream mechanisms and potentially druggable Notch effectors in TNBC CSCs are largely unknown. We studied the role of the AKT pathway and mitochondrial metabolism downstream of Notch signaling in TNBC CSC from cell lines representative of different TNBC molecular subtypes as well as a novel patient-derived model. We demonstrate that exposure of TNBC cells to recombinant Notch ligand Jagged1 leads to rapid AKT phosphorylation in a Notch1-dependent but RBP-Jκ independent fashion. This requires mTOR and IKKα. Jagged1 also stimulates mitochondrial respiration and fermentation in an AKT- and IKK-dependent fashion. Notch1 co-localizes with mitochondria in TNBC cells. Pharmacological inhibition of Notch cleavage by gamma secretase inhibitor PF-03084014 in combination with AKT inhibitor MK-2206 or IKK-targeted NF-κB inhibitor Bay11-7082 blocks secondary mammosphere formation from sorted CD90^hi^ or CD44^+^CD24^low^ (CSCs) cells. A TNBC patient-derived model gave comparable results. Besides mitochondrial oxidative metabolism, Jagged1 also triggers nuclear, NF-κB-dependent transcription of anti-apoptotic gene cIAP-2. This requires recruitment of Notch1, IKKα and NF-κB to the cIAP-2 promoter. Our observations support a model where Jagged1 triggers IKKα-dependent, mitochondrial and nuclear Notch1 signals that stimulate AKT phosphorylation, oxidative metabolism and transcription of survival genes in PTEN wild-type TNBC cells. These data suggest that combination treatments targeting the intersection of the Notch, AKT and NF-κB pathways have potential therapeutic applications against CSCs in TNBC cases with Notch1 and wild-type PTEN expression.

## Background

Triple negative breast cancer (TNBC) is a heterogeneous group of clinically aggressive breast cancers, designated as pathologically negative for estrogen receptor (ER^−^), progesterone receptor (PR^−^), and human epidermal growth factor receptor 2 amplification (HER2^−^). TNBC patients have a high risk of recurrence and metastasis, and current treatment options remain limited (1). Mortality is generally due to the development of metastatic or locally recurrent disease resistant to standard chemotherapy and radiation. Increasing evidence suggests that cancer stem-like cells (CSCs), present at diagnosis or evolved during treatment through epithelial-mesenchymal transition (EMT) and/or clonal selection are the driving force of tumorigenesis, chemo/radioresistance and metastatic spread. Therefore, CSC-targeted therapies are keenly sought. There is strong evidence for the involvement of Notch signaling in TNBC (2–6). Expression of Notch1 and its ligand Jagged1 correlate with poor prognosis, and expression of Notch1 mRNA correlates with poor survival in recurrent TNBC (7–10). Gene expression studies have led to the classification of TNBC into six (TNBCtype) (11) or more recently, four molecular subtypes (TNBCtype-4) (12), presenting unique molecular portraits and differential response to chemotherapy (13). In particular, the basal-like 1 (BL1) and mesenchymal stem-like (MSL, now merged with the Mesenchymal subtype) subtypes of TNBC are characterized by dysregulation of Notch modulators Lunatic Fringe (LFng) or Manic Fringe (MFng) and elevated expression of Notch1 (14–17). There is strong evidence supporting key roles of different Notch paralogs in breast CSCs (18–22, 23). However, key knowledge gaps prevent the successful targeting of this pathway in TNBC CSCs. In particular, the downstream mechanisms and potentially druggable Notch effectors in TNBC CSCs are largely unknown.

Notch signaling, an evolutionarily conserved cell fate determination pathway, is involved in multiple aspects of tumor biology, from angiogenesis to CSC maintenance to tumor immunity (4, 23). Humans and rodents express five canonical transmembrane Notch ligands [Delta-like ligand 1 (DLL1), DLL3, and DLL4, and Jagged 1 and Jagged 2], of which DLL3 serves a negative regulator (24) and four transmembrane Notch receptors (Notch1-4) (25–27). The interaction between a ligand on the surface of one cell and a receptor on an adjacent cell initiates a two-step proteolytic cleavage of the receptor. The first step is carried out by a disintegrin and metallo-proteinase (ADAM), generally ADAM10 (or ADAM17 in ligand-independent cleavage), and the second is mediated by γ-secretase, a multi-subunit enzyme complex which consists of Nicastrin, Presenilin, anterior pharynx-defective-1 (APH-1) and APH-2 as reviewed in (28). This sequential cleavage of Notch receptors results in release of a Notch-intracellular domain (NIC), which interacts with the CSL (CBF-1/Suppressor of Hairless/LAG-1) transcriptional regulator in the nucleus to de-repress transcription of target-genes. These in turn regulate cell-fate decisions, including differentiation, cell-cycle progression and survival. Post-translational modifications of Notch receptors can modulate their affinity for ligands as well as the intracellular half-lives of NIC (23, 29). Several classes of Notch-pathway inhibitors have been developed for clinical/pre-clinical investigation, including γ-secretase inhibitors (GSIs), monoclonal antibodies to Notch ligands or Notch receptors, monoclonal antibodies to Nicastrin, and Notch decoys (4). However, monotherapy targeting of CSC is not likely to achieve sustained therapeutic efficacy, due to signal redundancy, phenotypic plasticity and clonal evolution (4). Rational, mechanism-based combination of these agents with inhibitors of pathways downstream or upstream of Notch in specific subtypes of CSC are most likely to deliver sustained effectiveness while preventing dose-limiting toxicities.

In addition to canonical Notch signaling, a number of non-canonical (CSL-independent) Notch signals have been described in oncogenesis and inflammation (30–33). We and others have described activation of NF-κB via the IKK signalosome by Notch1 in T-ALL, cervical cancer and colorectal cancer (34–36). In cervical cancer (35) as well as ER^+^ breast cancer cells (37), respectively, Notch1 activates NF-κB and ERα-dependent transcription and triggers recruitment of IKKα to the chromatin. Activation of the AKT pathway downstream of Notch through suppression of PTEN expression and other mechanisms has been described in thymocytes, T-ALL, and mature T-cells (32, 38, 39). Association of Notch1 with the mitochondria has also been observed in glioma stem cells (31), T-cells (40), and macrophages (41). Bhola et al. (6) have shown that treatment of TNBC with either PI3K or mTORC1/2 inhibitors results in drug-resistant, Notch-dependent CSC. However, it remains unknown how Notch can overcome inhibition of the PI3K pathway and whether non-canonical Notch signaling contributes to this phenomenon.

In this study, we describe twin non-canonical mechanisms downstream of Jagged-1-mediated Notch1 activation that trigger AKT phosphorylation, mitochondrial metabolism and NF-κB activation in TNBC cells and CSC. Both pathways require IKKα, a potentially druggable kinase. We observed co-localization of Notch1 with mitochondria and metabolic activation in TNBC cells upon Jagged-1-mediated Notch1 activation. We found increased Notch1 expression, AKT phosphorylation and mitochondrial metabolism in mammospheres derived from TNBC cells. Pharmacological inhibition of Notch cleavage by gamma secretase inhibitor PF-03084014 in combination with AKT inhibitor MK-2206 or IKK inhibitor Bay11-7082 inhibits secondary mammosphere formation from CD90^hi^ or CD44^+^CD24^low^ (CSCs) sorted cells in TNBC cell lines as well as PDX-derived secondary mammosphere formation in a patient-derived model. Our data suggest that: (1) activation of AKT and mitochondrial metabolism is a consequence of non-canonical Notch activity in TNBC CSCs and (2) combination strategies targeting Notch and PI3K/AKT or IKK/NF-κB have potential therapeutic applications in targeting TNBC CSCs.

## Methods

### Cell Culture and Western Blotting

All TNBC cell lines were purchased from ATCC and maintained in DMEM or RPMI medium with 10% fetal bovine serum according to ATCC guidelines. For Western blots, cells were collected and suspended in RIPA buffer containing 1 mM PMSF, 1 mM sodium orthovanadate (Santa Cruz Biotechnology, SCB) and 1 mM Protease and Phosphatase Inhibitor Cocktail (Thermo Scientific). Protein concentration was determined by Bio-Rad protein assay. Equal amounts of protein were analyzed by 7.5% Criterion TGX Precast Gels (Bio-Rad) and transferred to PVDF membranes (Immobilon-FL Transfer Membrane, Millipore). Odyssey blocking buffer (LI-COR) was used to block non-specific binding sites and then membranes were incubated with primary antibodies to evaluate the expression of Notch1 (C-20-R), Notch4 (H-225), pIKKα (Thr 23), Total AKT (H-136), RBP-J (CSL), and β-actin (AC-15) (Santa Cruz Biotechnology); IKKα and pAKT (Ser473, D9E) (Cell Signaling Technology). After incubation with specific secondary antibodies (Goat anti-mouse 680RD or Goat anti-Rabbit 800CW; LI-COR), protein expression was detected using LI-COR Odyssey imaging system.

### Metabolic Flux Analysis (Seahorse)

XF-24 Extracellular Flux Analyzer (Seahorse Bioscience) was used to evaluate Oxygen consumption rate (OCR) and extracellular acidification rate (ECAR) in MDA-MB-231 (Mesenchymal-like, MSL) cells and in mammospheres generated from them. For OCR, cells were analyzed under basal conditions and in response to 1 μM oligomycin, 1 μM p-trifluoromethoxy-carbonylcyanide-phenylhydrazone (FCCP), 0.1 μM rotenone and 0.1 μM antimycin A (Sigma-Aldrich) using Mito-Stress test according to the manufacturer protocol (Agilent Technologies).

### SiRNA and DN-MAML Transfection

MDA-MB-231 cells were transiently transfected with non-targeting scrambled siRNA, RBP-Jκ siRNA, Notch1 siRNA, or IKKα siRNA (Santa Cruz Biotechnology) as per the standard manufacturer's protocol. Briefly, siRNA was mixed to siRNA Transfection Reagent (SCB) in siRNA Transfection Medium (SCB). After 30 min incubation at room temperature this mixture was added to cells for 6 h. Complete Medium (2 × FBS) was added to cells and 48 h after transfection cells were harvested. The expression levels of target proteins was determined by Western blot as described above.

MDA-MB-231 cells were transiently transfected a with dominant negative form of MAML1 (DN-MAML1) as we described earlier (37). Briefly, cells were transfected with Xfect Transfection Reagent (Clontech) according to the manufacturer's instructions protocol.

### Dual Luciferase Assay

MDA-MB-231 cells were transfected with scrambled siRNA or Notch1 (N-1) siRNA (Santa Cruz Biotechnology) or an expression plasmid encoding intracellular Notch1 (N1-IC) as described (35, 37). Twenty-four hours later, cells were transfected with NF-κB-luciferase reporter plasmid (NF-κB-Luc) and pTK-Renilla luciferase plasmid as internal control. The effects of Notch signaling modulation on NF-κB transcriptional activity were assessed by monitoring NF-κB promoter-derived luciferase activity. Luciferase assays (Dual-Luciferase Assay System, Promega) were performed according to the manufacturer's protocol. Luciferase activity was quantified by a luminometer (Veritas, CA) and firefly luciferase relative light emission units were normalized against Renilla luciferase activity. Relative luciferase activity was calculated considering 1.0 as the activity of cells transfected with empty vector.

### Quantitative Chromatin Immunoprecipation (CHIP)

Quantitative ChIP analysis was carried out as described (37, 42). Briefly, MDA-MB-231 cells were crosslinked with 1% formaldehyde, lysed in nucleus lysis buffer (1% SDS, 10 mM EDTA, 50 mM Tris-HCl, pH 8.1) and sonicated on ice at 95% total power for six cycles of 12 pulses each to achieve complete fragmentation of the DNA. These conditions were optimized in pilot experiments to achieve optimal fragmentation. After centrifugation, supernatant was collected and then IP solution was prepared by adding 9 volumes of dilution buffer (10 mM Tris-HCl, pH 8.1, 150 mM NaCl, 1 mM EDTA, 0.01% SDS, and 1% TritonX-100) to 1 supernatant volume. The IP solution was precleared with Salmon Sperm DNA/Protein G Agarose-−50% Slurry (Upstate Biotechnology, VA) for 1 h at 4°C with agitation. Following preclearing, the IP solution was incubated with the indicated antibodies (Notch1, p50, p65, and IKKα) overnight. The next day, immune complexes were recovered by incubation with Salmon Sperm DNA/Protein G Agarose-−50% Slurry for 2 h with gentle rocking. Agarose beads were pelleted by centrifugation and washed for 5 min each at 4°C with rocking with the following buffers: one wash in Low Salt Immune Complex Wash Buffer (0.1% SDS, 1% Triton X-100, 2 mM EDTA, 150 mM NaCl, 20 mM Tris-HCl (pH 8.0), one wash in High Salt Immune Complex Wash Buffer (0.1% SDS, 1% Triton X-100, 2 mM EDTA, 500 mM NaCl, 20 mM Tris-HCl (pH 8.0), one wash in LiCl Wash Buffer (0.25 M LiCl, 1% IGEPAL CA-630, 1% sodium deoxycholate, 1 mM EDTA, 10 mM Tris-HCl (pH 8.0), and two washes in TE buffer (pH 8.0). After final wash, the TE buffer was taken out by pipetting to ensure complete removal. Freshly prepared Elution Buffer (1% SDS, 0.1 M NaHCO_3_) was added to the beads at room temperature. Immune complexes were eluted by rocking for 15 min and collected. Elution was repeated as described, and Proteinase K (Invitrogen, CA) was added to each sample. Samples were incubated overnight at 65°C to break the formaldehyde crosslinks. The next day, DNA was recovered using the QIAquick PCR purification kit following manufacturer's instructions. Eluted DNA was analyzed by quantitative real-time PCR with the indicated primer pairs. The amounts of products were determined relative to a standard curve generated from a titration of input chromatin (37). Primers designed to amplify the NF-κB-site on cIAP2 promoter were: (Forward) 5- TGTGTGGTTATTACCGCTGG-3 and (Reverse) 5-GCGAGTCTCACGCTGTCTTT-3; primers for NF-κB-site on A20 promoter were: (Forward) 5-CTGCAGAAAAACAACTGCGA and (Reverse) 5-GTGAGTCACCTGGGCATTTC-3. PCR was also performed with primers for α-globin gene (Forward) CCAGCCTTATCCCAACCATA, (Reverse): TATCATGCCTCTTTGCACCA as an internal control.

### Immunofluorescence and Confocal Microscopy

Immunofluorescence analysis was performed on MDA-MB-231 and MDA-MB468 cells to evaluate the co-localization Notch1 and mitochondria. Cells were grown on Lab-Tek chamber slides (Nunc) and then stained with a mitochondrion-selective probe (MitoTracker-Red, 200 nM) according to the manufacturer's protocol (Invitrogen). Following a PBS wash, cells were fixed in 2% PFA followed by blocking in 5% normal goat serum. Notch1 antibody (C-20, 1:50 dilution) (Santa Cruz Biotechnology) was added for overnight incubation at 4°C. Following PBS wash, AF-488 conjugated secondary antibody (1:200 dilution; Invitrogen) was for detection. Slides were then washed in PBS, mounted with DAPI (Vector Laboratories), and visualized using a Confocal Microscope (Olympus FV1000). The colocalization indexes Pearson's and Manders' coefficients were calculated using the ImageJ colocalization analysis plugin. The portions of the cell occupied by Notch1 and mitochondria were calculated as the ratio of the fluorochromes' areas (visualized by AlexaFluor488 and mito-Red, respectively) to the entire cell area (visualized by blue masking). After background subtraction, the mean intensity of each fluorescence was used as a threshold to obtain binary images on which the different areas were measured (*n* = 20 cells per group were analyzed).

### Establishment of TU-BCx-2K1 Patient-Derived Cell Line

The triple negative patient-derived tumor, designated as 2K1, was acquired in collaboration with the Louisiana Cancer Research Consortium Biospecimen Core and processed in compliance with NIH regulations and institutional guidelines, and approved by the Institutional Review Board at Tulane University. These experiments were performed at Tulane University. 2K1 was established and propagated in immunocompromised SCID/Beige mice. SCID/Beige mice *(*CB17.Cg-*Prkdc*^*scid*^*Lyst*^*bg*^/Crl*)* were purchased from Charles River and are used to prevent rejection of the xenografted human tumors. The autosomal recessive SCID (Prkdc^scid^) mutation results in severe combined immunodeficiency affecting both the B and T lymphocytes. The autosomal recessive beige (Lyst^bg^) mutation results in defective natural killer (NK) cells. A tumor explant (3 × 3 mm^2^) was plated in a 6-well plate with DMEM supplemented with 10% FBS, non-essential amino acids (NEAA), MEM amino acids, anti-anti (100 U/mL), sodium pyruvate and porcine insulin (1 × 10^−10^ mol/L) at 37°C in humidified 5% CO_2_. TU-BCx-2K1 was generated from cells that adhered to the dish weeks after the explant was plated.

### Mammospheres Assays

Primary mammospheres were generated from MDA-MB-231 (Mesenchymal-like, MSL) or MDA-MB-468 (Basal-like, BL1) cells using MammoCult Human Medium Kit (STEMCELL Technologies). Briefly, after 7 days incubation on ultra-low attachment 6-well plate (Corning) in MammoCult growth medium, P1 mammospheres were collected by gentle centrifugation and then trypsinized for single cell suspension. Cells (10,000/well) were plated on ultra-low attachment 6-well plate (Corning) with complete MammoCult growth medium in the presence of GSI PF-03084014 (5 μM) or AKT inhibitor MK-2206 (5 μM) or IKK-targeting NF-κB inhibitor Bay11-7082 (1 μM) (Selleckchem, Texas) twice/week. Following 7 days of incubation, mammospheres (>100 μm) were counted manually using a Nikon Eclipse Microscope. For CSCs enrichment, CD44^+^CD24^low^ and CD90^+^ cells were sorted from MDA-MB-231 cells using LSRII Flow cytometer (BD Biosciences). Sorted cells were plated for mammosphere formation, followed by treatments with GSI, AKT inhibitor or NF-κB inhibitor as described.

### PDX Mammosphere Culture

Mammospheres were cultured in low suspension media composed of DMEM/F-12 media supplemented with B-27, penicillin-streptomycin, fibroblast growth factor (FGF) and epidermal growth factor (EGF) (Invitrogen, Carlsbad, CA). Mammospheres were created by plating TU-BCx-2K1 PDX cells (50,000) in low suspension DMEM/F-12 media supplemented with FGF and EGF (20 ng/mL each) in low-attachment 6-well plates (Thermo Fisher Scientific, Waltham MA). Mammospheres were treated with GSI PF-03084014, AKT inhibitor (MK-2206) or IKK inhibitor, Bay11-70820 (Selleckchem, Texas) as described earlier.

## Results

### Non-canonical Notch Signaling Activates AKT Phosphorylation in PTEN-Wild Type TNBC Cells of Some Molecular Subtypes

**Figure 1:**
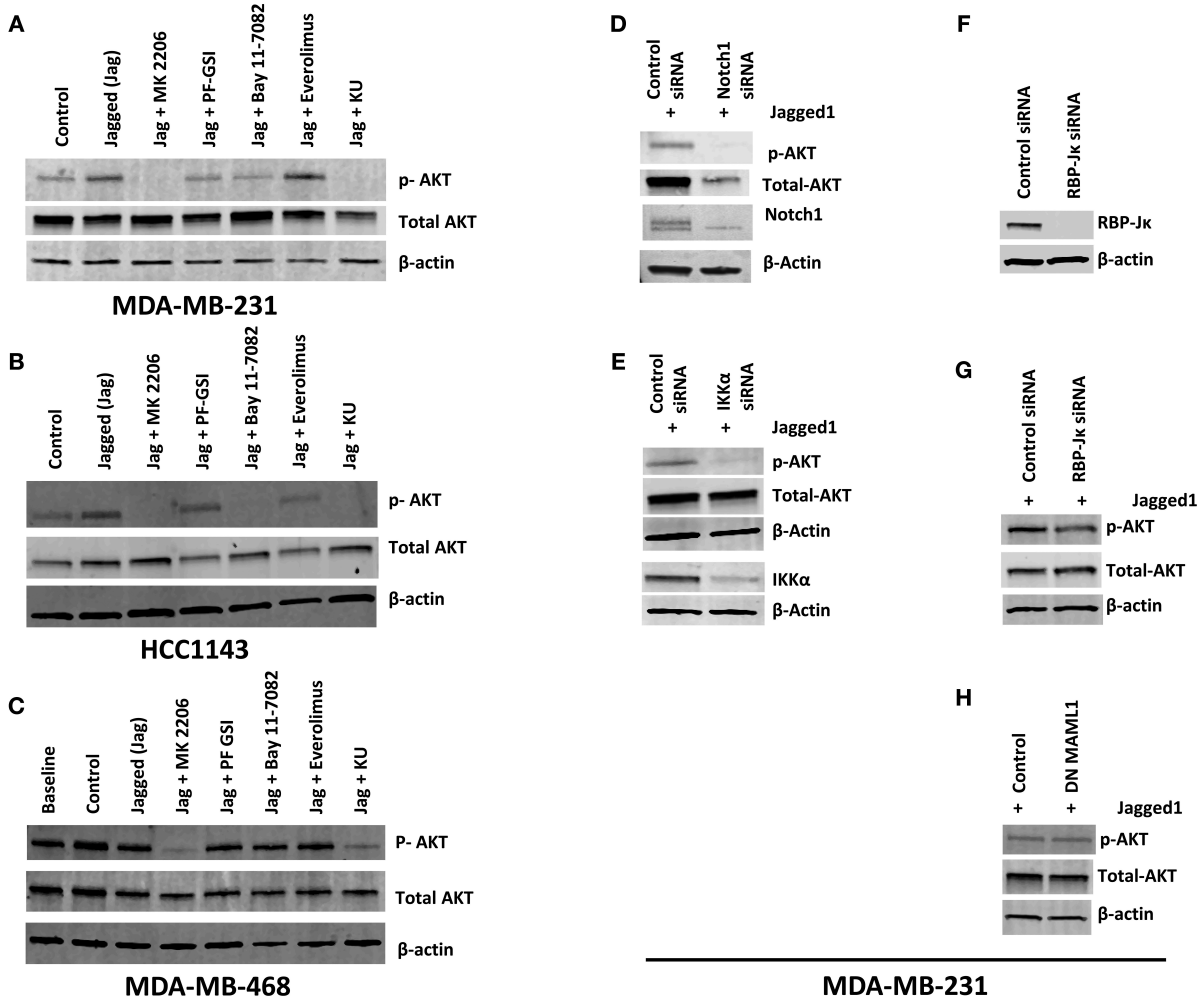
Notch activation induces AKT phosphorylation independently of RBP-Jκ but dependent on IKKα in PTEN-wt TNBC cells. **(A,B)** MDA-MB-231 (MSL, PTEN-wt) and HCC1143 (BL1, PTEN-wt) cells were plated on gelatin (0.2%) (Control) or human recombinant Jagged1 (1 μg/ml) in gelatin (Jagged) coated plates in the presence of indicated drugs (AKT inhibitor MK-2206, 5 μM), GSI PF-03084014 (5 μM), BAY11-7082 (IKK inhibitor, 5 μM), Everolimus (mTORC1 selective inhibitor, 5 μM), and KU-0063794 (dual mTORC1/mTORC2 inhibitor, 5 μM) for an hour. Western blotting was carried out using whole cell lysates. **(C)** Similarly, MDA-MB-468 (BL1, PTEN null) cells were plated on control or Jagged1 coated plates for an hour in the presence of indicated drugs and western blot was carried out using whole cell lysates. **(D,E)** MDA-MB-231 cells were transfected with control siRNA or Notch1siRNA or IKKα siRNA. Forty-eight hours following transfection, equal number of cells were plated on Jagged coated plates for an hour and AKT activation was measured by Western Blot. **(F,G)** RBP-Jκ was silenced in MDA-MB-231 cells using siRNA. RBP-Jκ silenced and or control cells were plated on Jagged1 coated plates for an hour and phosphorylation of AKT was determined using Western Blot. **(H)** MDA-MB-231 cells were transfected with a dominant negative form of MAML1 (DN-MAMAL1) or control vector. Cells were then plated on Jagged1 coated plates for an hour and phosphorylation of AKT was determined using Western Blot.

Numerous studies suggest that Notch1 activates or is required for activation of the AKT pathway in various systems (43–45). The AKT signaling pathway is involved in multiple cellular functions including promoting cell growth, increasing glucose uptake and oxidation, cell cycle progression and cell survival (46, 47). In TNBC, resistance to PI3K or mTORC1/2 inhibitors is mediated by Notch through unknown mechanisms (6). Therefore, we investigated whether Notch1 causes AKT activation in TNBC cells. Initial experiments were performed in the co-culture system we used to dissect the crosstalk of Notch1 with ERα (37). Co-culture of MDA-MB-231 cells with LTK-JAG fibroblasts does induce rapid phosphorylation of AKT (S473) as well as IKKα (S180) (Supplemental Figures 1A,B). However, the complexity of the cellular system used prevented us from determining the mechanism of this effect. Notch1 activation in a fibroblast-free system using EDTA (48, 49) in MDA-MB-231 cells was accompanied by S473 AKT and S180 IKKα phosphorylation within minutes, which was suppressed by Notch1 knockdown (Supplemental Figure 1C). Exposure to recombinant Jagged1 for 1 h induced phosphorylation of AKT at S473 in MDA-MB-231 cells (Figure 1A). The kinetics of activation in this system was slower than with EDTA, presumably because it requires re-attachment of suspended cells and productive interaction with Jagged1. In this system, induction of IKKα phosphorylation was not consistently observed, perhaps due to the slower kinetics. Jagged-induced AKT phosphorylation was inhibited by a gamma secretase inhibitor (GSI PF-3084014) and completely abrogated by dual mTORC1/mTORC2 inhibitor KU-0063794. Conversely, mTORC1-selective inhibitor Everolimus had no effect. Interestingly, IKK inhibitor Bay11-7082 was as potent as GSI, suggesting that in this system, IKKα or the IKK signalosome is upstream of AKT activation (Figure 1A). Identical results were obtained in HCC1143 cells (Basal-like 1, PTEN wild type) (Figure 1B) but not in MDA-MB-468 (Basal-like 1, PTEN-null) (Figure 1C). Knockdown of Notch1 suppressed AKT phosphorylation following Jagged1-activation (Figure 1D). Interestingly, Notch1 knockdown also reduced total AKT levels. We do not know the mechanism of this effect. However, mTORC2-dependent phosphorylation of AKT at T450 has been proposed to control AKT protein stability (50). Whether Notch1 regulates the T450 phosphorylation of AKT remains to be determined. We and others have shown that IKKα physically interacts with Notch1 and functions downstream of Notch1 in cervical cancer (35), T-ALL (34), ER^+^ breast cancer (37) and colorectal cancer (36, 51). Xu et al. (52) have shown that IKKα and IKKβ cause AKT activation by physical interaction and direct phosphorylation of mTORC2 subunit Rictor in a variety of cell lines. Babaev et al. (53) have recently shown that genetic or pharmacological inhibition of IKKα causes reduced mTORC2 activity and AKT phosphorylation in macrophages, which results is increases sensitivity to apoptosis. Given the effect of Bay11-7082 on AKT phosphorylation, we investigated whether IKKα mediates Notch-induced AKT phosphorylation in our system. IKKα knockdown decreased AKT phosphorylation in recombinant Jagged1-stimulated MDA-MB-231 cells (Figure 1E). This indicates that the expression of IKKα is required for this effect of Notch1. Complete knockdown of RBP-Jκ in MDA-MB-231 cells had no effect on Jagged1-induced rapid AKT activation (Figure 1G). Similar results were obtained when we transfected MDA-MB-231 cells with dominant negative MAML1 (DN MAML1) (Figure 1H), which lacks the domain responsible for co-activator recruitment but binds to Notch1 and CSL. Taken together, these results indicate that Jagged-induced rapid AKT activation is a non-canonical phenomenon, which is IKKα-dependent and CSL/MAML1-independent. Our results do not rule out longer-term effects on AKT signaling mediated by canonical transcriptional effects.

Next, we extended our observations to other TNBC cell lines representative of different molecular subtypes (11, 12). MDA-MB-231 cells are representative of the Mesenchymal/Stem-like (M/MSL) subtype, and are PTEN wild type. In these cells, AKT phosphorylation was inhibited by GSI and abolished by Bay11-7082 (Figure 1A). Conversely, in PTEN-null, Basal-like 1 (BL1) MDA-MB-468 cells, AKT phosphorylation was insensitive to either GSI or IKKα inhibition (Figure 1C). PTEN-mutant M/MSL BT549 cells were sensitive to IKKα inhibition but not GSI (Supplemental Figure 2). In CDKN2A-mutant BL2 HCC1806 cells, AKT phosphorylation was sensitive to IKKα inhibition but not GSI (Supplemental Figure 2). Luminal androgen receptor (LAR) subtype TNBC cells MDA-MB-453 were moderately sensitive to IKKα inhibition and GSI (Supplemental Figure 2). AKT phosphorylation in all the cell lines tested was sensitive to dual mTOR inhibitor KU-063794 but not Everolimus (mTORC1 inhibitor), consistent with the role of mTORC2 in catalyzing S473 AKT phosphorylation. These observations indicate that the pathway we identified is active in some but not all TNBC cells, depending on PTEN status and possibly other molecular features.

### Jagged1 Regulates Cellular Bioenergetics in a Notch1 and IKKα Dependent Fashion

**Figure 2:**
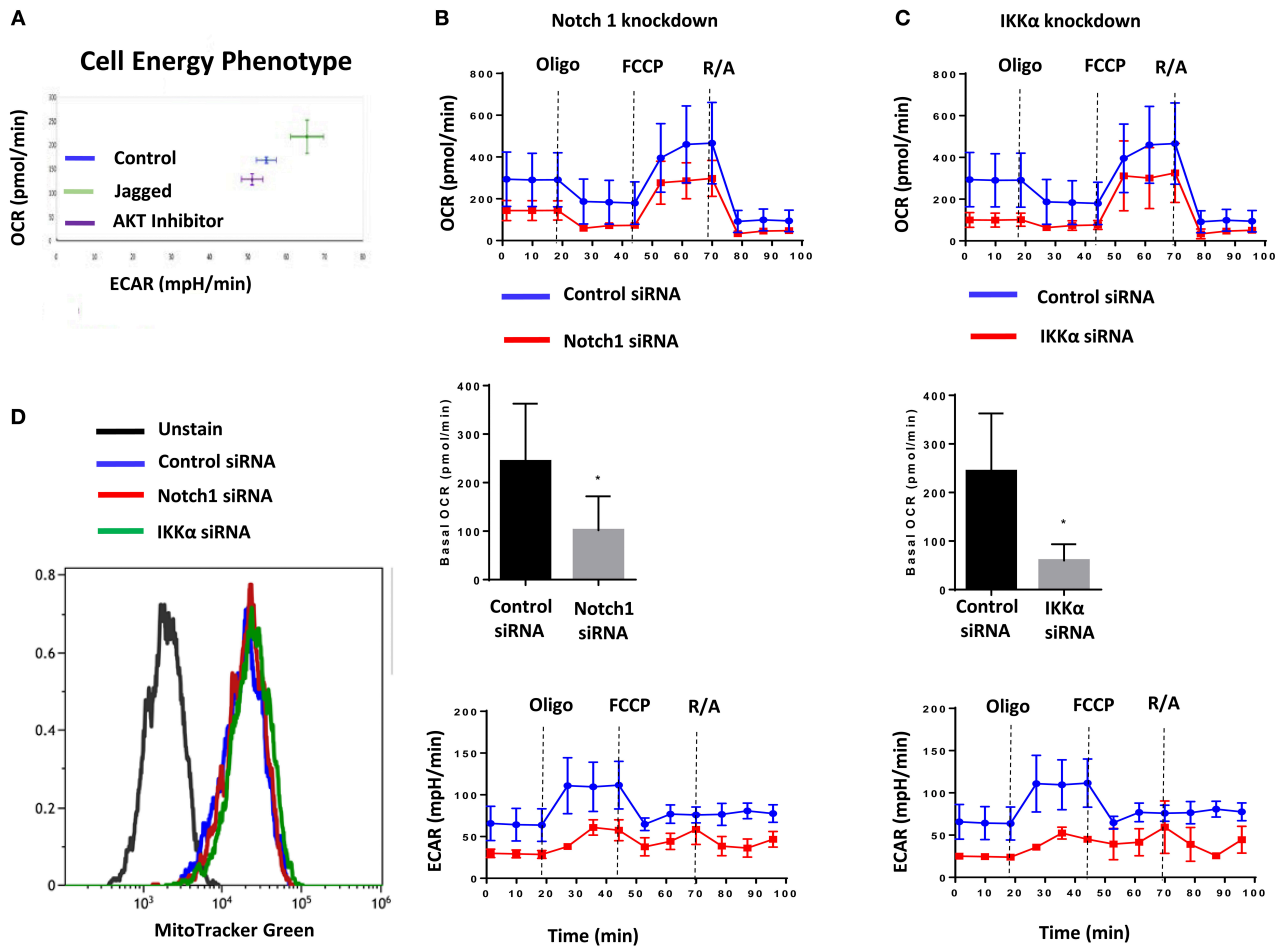
Jagged1 regulates Cellular Bioenergetics in Notch and IKKα dependent fashion. **(A)** MDA-MB-231 cells were plated on control or Jagged1 coated XF24 Cell Culture plate in the presence or absence of AKT inhibitor (MK-2206, 5 μM). Cell energy phenotype profile, mitochondrial respiration as Oxygen Consumption Rate (OCR) and fermentation as indicated by Extracellular Acidification Rate (ECAR) was measured using a Seahorse Analyzer as described in the Methods section. **(B,C)** MDA-MB-231 cells were transfected with control siRNA or Notch1 siRNA or IKKα siRNA. Forty eight hours following transfection, equal number of live cells were plated on Jagged1 coated XF24 cell culture plate and analyzed for OCR and ECAR by Seahorse Analyzer. Basal OCR was the difference between the OCR before Oligomycin and after Rotenone & Antimycin A (R/A). **(D)** Control and Notch1 or IKKa siRNA transfected cells were stained with 50 nM MitoTracker Green FM (Invitrogen) to detect Mitochondrial Mass by Flow Cytometer according to the manufacturer protocol. Data were analyzed using Beckman Coulter Kaluza Analysis Software.

The AKT pathway is a major driver of glucose uptake and aerobic metabolism (54). Notch1 has been reported to stimulate glucose metabolism in thymocytes (55, 56) and MCF-7 cells (57). Therefore, we explored whether the Notch-AKT pathway we describe herein regulates cellular metabolism in TNBC cells using a Seahorse Analyzer. Jagged1-mediated Notch activation increased cellular bioenergetics in MDA-MB-231 cells as measured by OCR (Oxygen Consumption Rate) and ECAR (Extracellular Acidification Rate) (Figure 2A). Pharmacological AKT inhibition blocked both OCR and ECAR increases induced by Jagged1 (Figure 2A). Knockdown of Notch1 by siRNA decreased both OCR and ECAR under basal (Supplemental Figure 3A) and Jagged1-induced conditions (Figure 2B). To avoid artifacts due to inhibition of growth or survival by siRNA transfection, equal numbers of viable cells from control siRNA- or Notch1 siRNA-transfected cells were plated for Seahorse experiments. Similarly, IKKα knockdown decreased OCR and ECAR under both basal (Supplemental Figure 3B) and Jagged1-induced conditions (Figure 2C). Importantly, there was no change in the mitochondrial mass of Notch1 siRNA or IKKα siRNA-transfected cells (Figure 2D). Cumulatively, these findings support the hypothesis that Jagged1 regulates cellular bioenergetics in some TNBC cells through Notch1, IKKα, and AKT.

### Notch1 Co-localizes With Mitochondria in TNBC Cells

**Figure 3:**
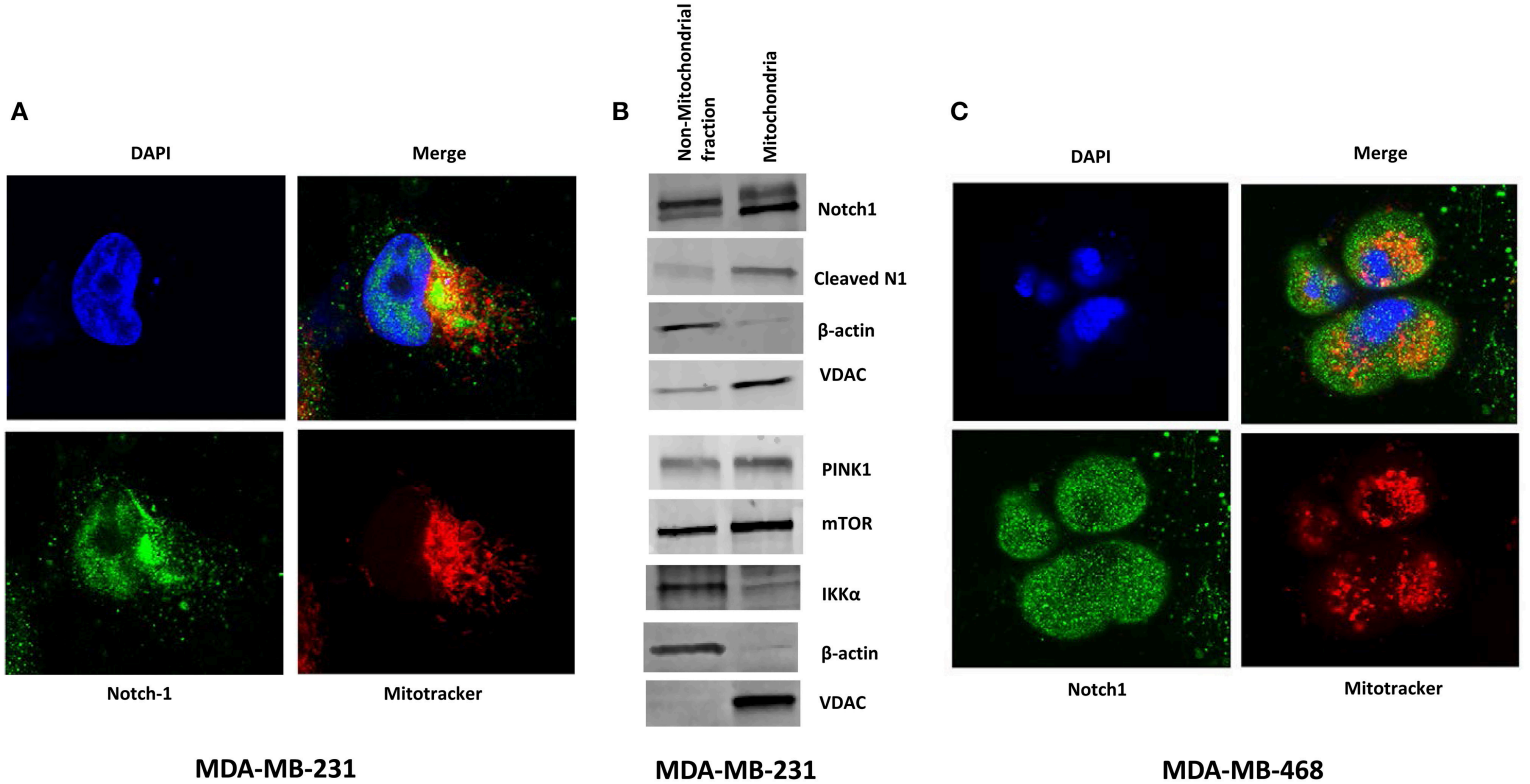
Notch1 co-localizes with Mitochondria in TNBC cells. **(A)** MDA-MB-231 cells were grown on Lab-Tek II chamber slides and stained with mitochondrial tracer (MitoTracker), Notch1 (C-20) antibody, and DAPI (nucleus) and co-localization of Notch and Mitochondrial was visualized by confocal microscopy. **(B)** Mitochondria were isolated form MDA-MB-231 cells using a Mitochondria Isolation Kit for cultured cells (Abcam). Protein expression (indicated) was determined from the mitochondrial lysate and the non-mitochondrial fraction by Western blotting. **(C)** Similarly MDA-MB-468 cells were stained with mitochondrial tracer (MitoTracker), Notch1 (C-20) antibody, and DAPI (nucleus) and co-localization of Notch1 and mitochondria was visualized using a Confocal microscope.

Perumalsamy et al. (40) first showed that Notch signaling promotes survival in T-cells via a non-nuclear AKT-mediated pathway that promotes mitochondrial integrity. The same group also showed that Notch1-IC physically interacts with Rictor/mTORC2 in T-reg cells (58). More recently, Lee et al. demonstrated in glioma stem cells and Drosophila neural stem cells that Notch1 activates the mTORC2/AKT pathway by associating with Rictor on the mitochondrial surface, via PINK1 (31). Association of Notch with the mitochondria has been described also in T- cells (40) and macrophages (41). One study suggests that Notch1 may even regulate the transcription of mitochondrial proteins (59). Thus, we sought to determine whether Notch1 associates with mitochondria in TNBC cells as well. Immunofluorescence staining of MDA-MB-231 cells revealed the presence of Notch1 in both nucleus and cytoplasm (Figure 3A). A significant fraction of the cytoplasmic Notch1 signal co-localized with mitochondria even under basal conditions (Figure 3A). We found similar results in MDA-MB-468 cells as well (Figure 3C). We analyzed the Menders and Pearson's co-localization coefficients using the ImageJ co-localization analysis plugin. The Pearson's co-localization coefficients in MDA-MB-231 and MDA-MB-468 cells were 0.408 ± 0.019 and 0.368 ± 0.027, respectively (*P* < 0.01), while the Manders' coefficients were 0.629 ± 0.016 and 0.570 ± 0.024, in MDA-MB-231 and MDA-MB-468 cells, respectively (*P* < 0.01), supporting the co-localization of Notch with mitochondria. We then analyzed a purified mitochondrial fraction of MDA-MB-231 cells by Western blotting. Cleaved Notch1 (Notch1-IC) was enriched in the mitochondrial fraction (Figure 3B), consistent with the findings of Lee et al. (31). PINK1, mTOR and IKKα were also found in purified mitochondrial fractions along with other cellular fractions (Figure 3B). Future studies will determine whether this mitochondrial localization is required for Notch1 to activate mTORC2-AKT.

### MDA-MB-231 Mammospheres Have Increased Oxidative Metabolism and Are Sensitive to GSI, AKT, or IKK Inhibition

Mitochondria are indispensable for cellular respiration and aerobic metabolism. Although aerobic glycolysis has been long recognized as a metabolic feature of many cancer cells, breast CSC have been shown to rely on enhanced oxidative metabolism, contrary to their more differentiated counterparts (60). Hence, we sought to determine whether the pathway we describe herein is active and therapeutically targetable in breast CSC.

**Figure 4:**
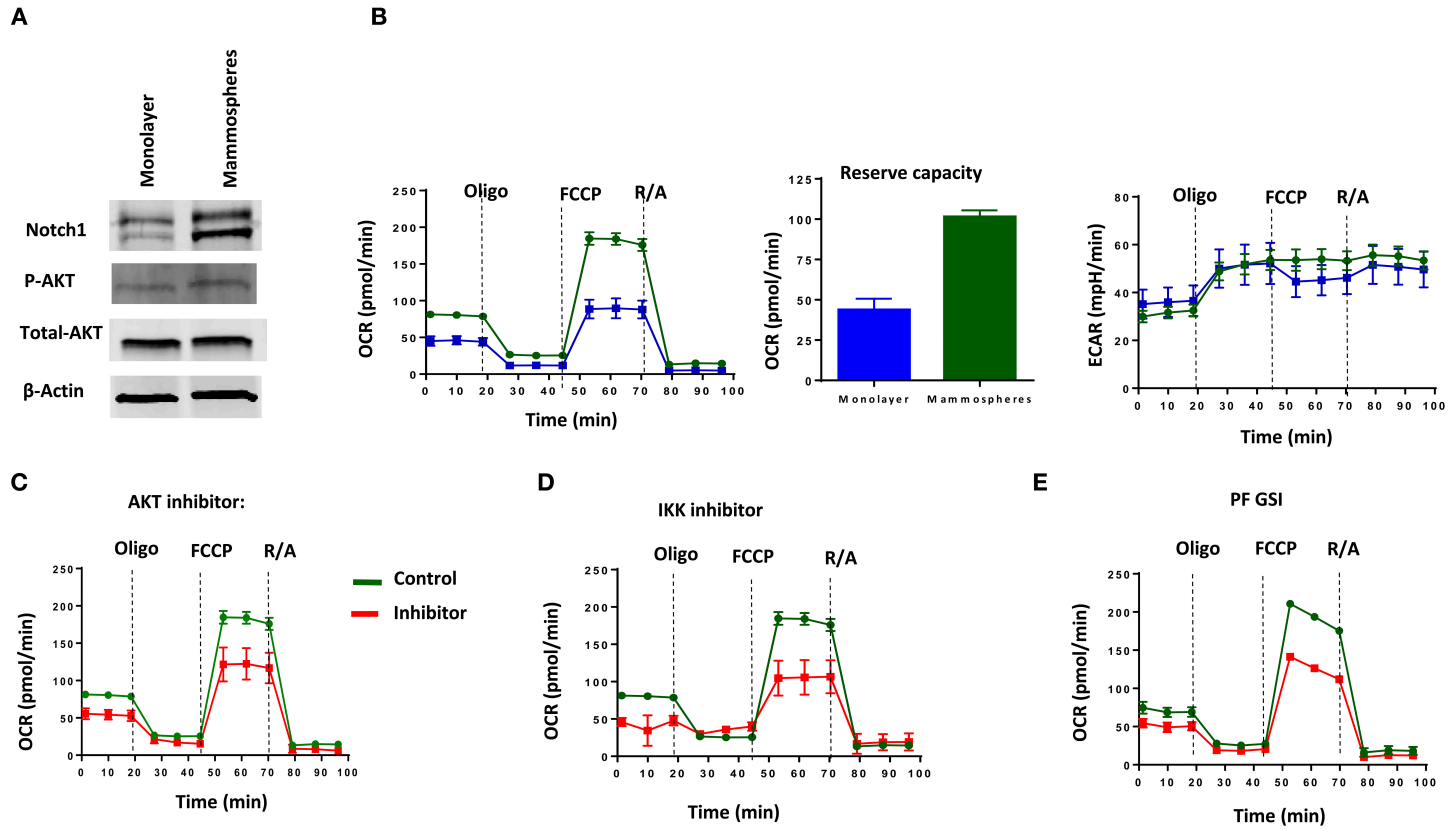
MDA-MB-231 Mammospheres have increased oxidative metabolism, which is sensitive to GSI, AKT inhibition or IKK inhibition. Mammospheres were enriched from MDA-MB-231 cells using human Mammocult media as per the manufacturer's protocol (STEMCELL Technologies). **(A)** Expression of Notch1 and AKT phosphorylation was determined in mammospheres and monolayer cell lysates by Western blotting. **(B)** OCR including Reserve Capacity and ECAR were compared between mammospheres and monolayer cells using a Seahorse Analyzer. Mammospheres were treated with **(C)** AKT inhibitor (MK-2206, 5 μM); **(D)** IKK inhibitor (Bay11-7082, 1 μM) or **(E)** GSI PF-03084014 (PF, 5 μM) and then OCR and ECAR were measured using a Seahorse Analyzer.

Consistent with the literature (21, 61), we found increased Notch1 and pAKT in passage 1 (P1) mammospheres generated from MDA-MB-231 cells (Figure 4A). P1 mammospheres had increased basal and maximum mitochondrial respiration compared to adherent cells, but no significant differences in extracellular acidification rate (Figure 4B). Mammospheres also had increased reserve respiratory capacity compared to monolayer cells (Figure 4B), suggesting a switch toward oxidative metabolism. Pharmacological AKT inhibition with allosteric inhibitor MK-2206 or IKK inhibition with Bay11-7082 decreased both basal and maximum OCR (Figures 4C,D). We recently showed that among five different GSIs tested in clinical trials, all of which demonstrate anti-CSC activity, PF-03084014 had superior anti-mammosphere activity (62). PF-03084014 also decreased both OCR and ECAR (Figure 4E) in P1 mammospheres. These results suggest that Notch inhibition in combination with either AKT or IKK inhibition can be used to target mammosphere metabolism in Notch-dependent TNBC cells.

### GSI PF-03084014 Has Anti-CSCs Activity in Combination With an AKT Inhibitor or an IKK Inhibitor

**Figure 5:**
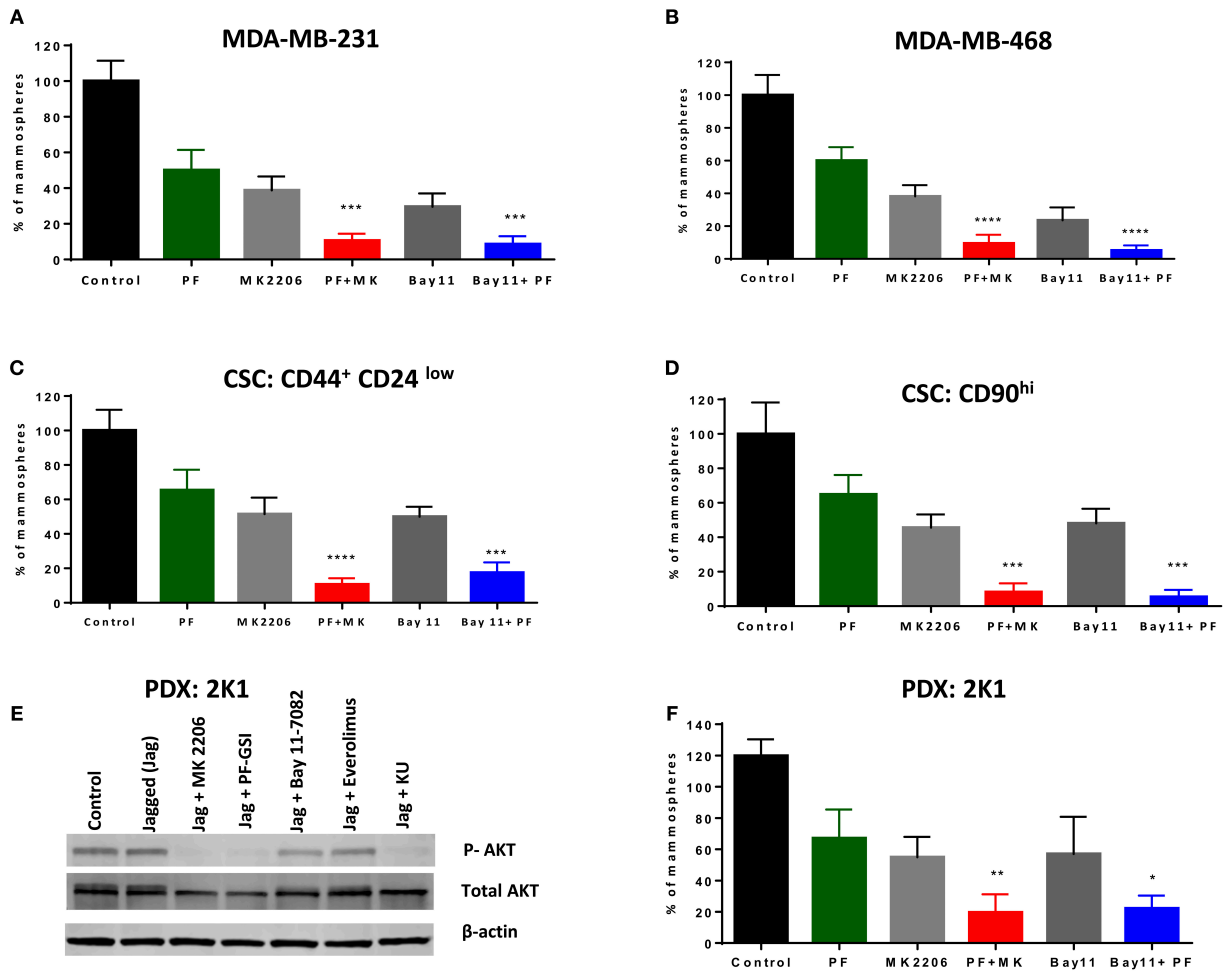
GSI PF-03084014 has anti-CSCs activity in combination with an AKT inhibitor or an IKK inhibitor. Mammospheres from MDA-MB-231 **(A)** or MDA-MB-468 cells **(B)** were treated with GSI PF-03084014 (PF, 5 μM) or AKT inhibitor MK-2206 (MK, 5 μM) or IKK inhibitor Bay11-7082 (Bay11, 1 μM) as single agents or in combinations including PF (5 μM) plus MK (5 μM) or PF(5 μM) plus Bay11(1 μM) for 1 week (twice per week treatment). Following incubation, mammospheres were counted using a Nikon microscope. Cancer stem like cells, (CD90^hi^ or CD44^+^CD24^low^) were sorted from MDA-MB-231 cells using a BD FACS Aria II as per the standard protocol. MDA-MB-231 sorted cells **(C)** CD90^hi^ or **(D)** CD44^+^CD24^low^ were cultured in Mammocult media for 1 week and then P1 mammospheres were treated as indicated and counted as described. **(E)** PDX:2K1cells were plated on control or Jagged1 coated plates for an hour in the presence of the indicated drugs: AKT inhibitor MK-2206 (5 μM), GSI PF-03084014 (5 μM), IKK inhibitor BAY11-7082 (5 μM), mTORC1 selective inhibitor Everolimus (5 μM), and dual mTORC1/mTORC2 inhibitor KU-0063794 (5 μM) for an hour, after which AKT phosphorylation (S476) was measured by Western blotting. **(F)** PDX mammospheres were enriched from PDX:2K1 cells as described above, and P1 PDX mammospheres were treated with indicated drugs and counted as described above. Data, mean ± SD; *P*-values: *P < 0.05; **P < 0.01; ***P < 0.001; ****P < 0.0001.

We sought to determine whether a GSI alone or in combination with AKT or IKK inhibition inhibits mammosphere growth. We have shown in the past that several GSIs inhibit mammosphere formation in patient-derived samples (21). As we recently determined that PF-030840414 has superior anti-mammosphere activity among the GSIs we tested (62), we evaluated this agent alone or in combination with AKT inhibitor MK-2206 or IKK inhibitor Bay11-7082. Equal numbers of P1 mammospheres were plated in the presence of GSI PF-03084014 (GSI-PF) alone or in combination with MK-2206 or Bay11-7082 for another 7 days. Each single treatment (GSI or AKT inhibitor or IKK inhibitor) results in a significant decrease in mammosphere formation (Figure 5A) but did not abolish mammosphere formation. However, the combination of GSI-PF with either an AKT or an IKK inhibitor significantly abrogated mammosphere growth (Figure 5A). This effect was not limited to MDA-MB-231 cells, as it was observed also in MDA-MB-468 cells (Figure 5B), although in these cells AKT phosphorylation is GSI-insensitive. These results suggest that the anti-mammosphere activity of GSI PF-03084014 is not exclusively due to inhibition of the IKKα-AKT pathway, but it is significantly increased when either kinase in inhibited. We obtained virtually identical results when we tested secondary mammospheres generated from CSCs flow-sorted based either on CD44^+^CD24^low^ (63) or CD90^hi^ (64) (Figures 5C,D). These data suggest that treatments with a GSI in combination with either an AKT inhibitor or an IKK inhibitor may be an attractive, mechanism-based anti-CSC strategy in some TNBCs.

### GSI (PF-03084014) in Combination With an AKT Inhibitor or an IKK Inhibitor Is Effective Against PDX-Derived Mammospheres

Patient-derived xenografts (PDX) are an evolving model for cancer experimental therapeutics, which is thought to more closely represent cancer clones existing in patients. To test whether the combination treatments we propose has activity in a patient-derived TNBC model, we developed a PDX model from a TNBC patient as described in Methods. A cell line was generated from PDX tissue (2K1T6) and tested as monolayer and mammospheres. These cells constitutively expressed Notch1 (Supplemental Figure 6A), like established TNBC cell lines. These PDX cells have constitutively active AKT phosphorylation which was not further increased by exposure to Jagged1, but was virtually abolished by GSI, AKT inhibitor MK-2206 or dual mTORC inhibitor KU-063794, and modestly inhibited by Bay11-7082 (Figure 5E). As we observed in TNBC cell lines, single agents GSI, AKT inhibitor or IKK inhibitor inhibited but did not abrogate mammospheres growth. GSI plus AKT inhibitor or GSI plus IKK inhibitor treatment drastically inhibited mammospheres growth in this PDX-derived model (Figure 5F; Supplemental Figure 6B). Taken together, these results reinforce the proposition that combinations of Notch inhibitors with AKT or IKK inhibitors deserve further investigation as anti-CSC treatments in TNBC.

### Notch1 Also Activates NF-κB-Dependent Transcription in an IKKα-Dependent Fashion in MDA-MB-231 Cells

**Figure 6:**
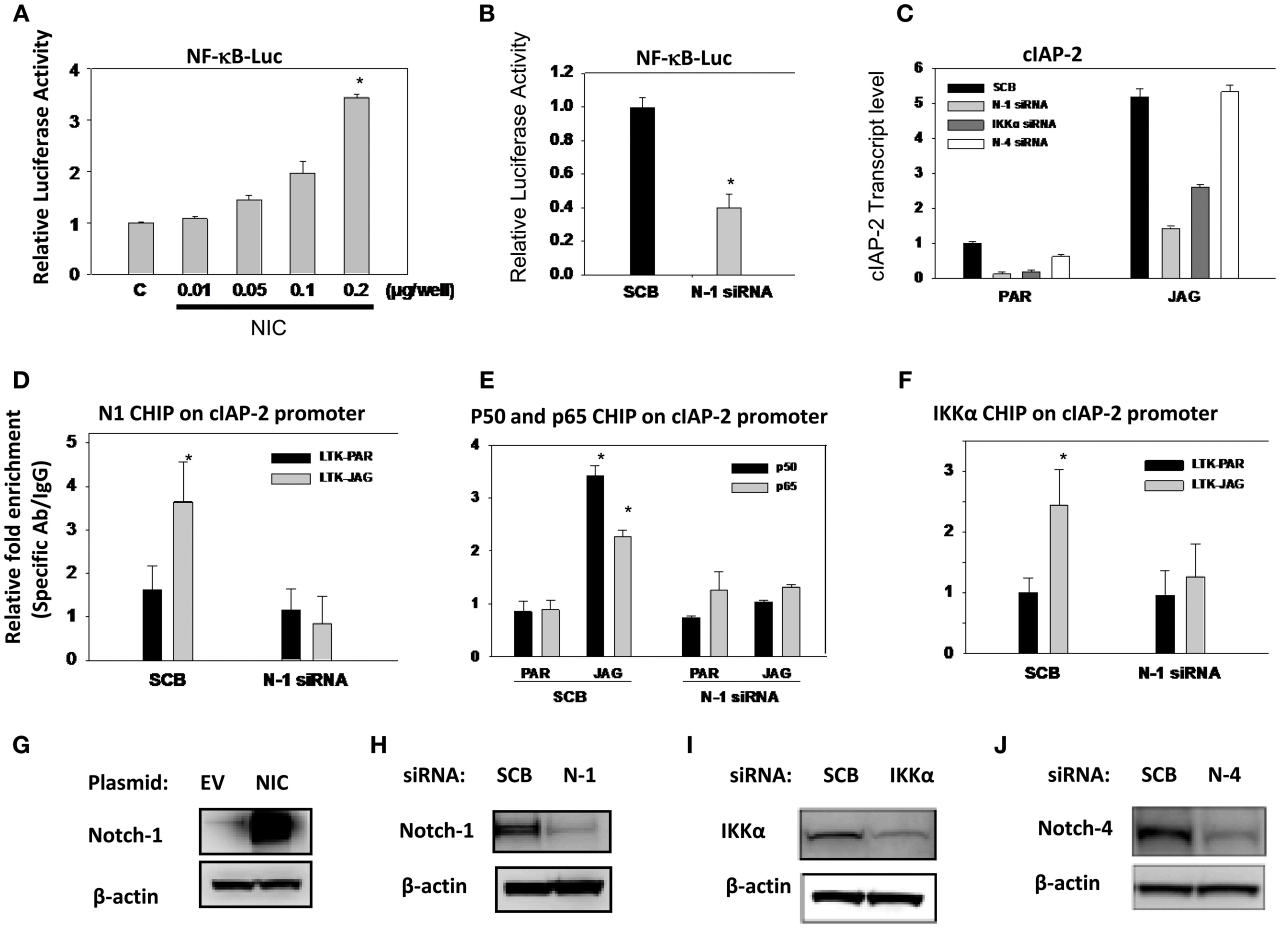
Notch1 activates NF-κB-dependent transcription in an IKKα-dependent fashion in MDA-MB-231 cells. **(A)** MDA-MB-231 cells were co-transfected with NF-κB luciferase reporter plasmid plus pTK-Renilla plasmid as well as an expression plasmid encoding intracellular Notch1 (NIC). The effects of Notch signaling modulation on NF-κB transcriptional activity were assessed by monitoring NF-κB promoter-driven luciferase activity. Relative luciferase activity was calculated considering 1.0 as the activity of cells transfected with empty vector. **(B)** Cells were transfected with scrambled siRNA (SCB) or Notch-1 (N-1) siRNA. Twenty-four hours later, cells were transfected with NF-κB-luciferase reporter plasmid plus pTK-Renilla luciferase plasmid as an internal controls. NF-κB transcriptional activity was assessed by monitoring NF-κB promoter-driven luciferase activity. **(C)** MDA-MB-231 cells were transfected with either scrambled siRNA (SCB), or Notch-1(N-1), Notch-4 (N-4), IKKα siRNA. Following transfection, cells were co-cultured with mouse fibroblast parental LTK cells (PAR) or LTK cells overexpressing Jagged-1 (JAG). Relative transcript levels were determined by RT-PCR. **(D,F)** MDA-MB-231 cells were transfected with scrambled (SCB) or Notch-1 siRNA (N-1 siRNA) and 48 h later co-cultured with PAR or JAG LTK cells. ChIP assays using specific antibodies were performed as described in the Methods section. ChIP data were expressed as relative fold enrichment of specific antibody over IgG control. Data were normalized to internal control: non-specific binding to a β-globin intron sequence. **(G–J)** Overexpression of Notch-1 and knocking down of Notch-1, Notch-4, and IKKα by siRNA were validated by Western Blotting.

Previously, we reported that Notch1 associated with IKKα and regulated NF-κB activity in cervical cancer cells (35). In the same paper, we showed that Notch-1 and IKKα associate with the promoter of apoptosis inhibitor c-IAP2 in CaSki cells and that in the presence of TNF-α, additional Notch-1 and IKKα are recruited at the c-IAP2 promoter. Subsequently, we showed that in luminal breast cancer cells, Notch1, and IKKα are recruited to ERα-responsive elements and activate ER-dependent transcription in an estrogen-independent fashion (37). Similarly, Espinosa et al., demonstrated that nuclear IKKα is a key mediator of the oncogenic effects of Notch1 in colorectal cancer (36, 51). Hence, we sought to determine whether in addition to AKT activation, IKKα also mediates nuclear NF-κB-dependent transcription in TNBC cells. We transfected MDA-MB-231 cells with a plasmid expressing the active form of Notch1 (Notch1-intracellular, N1-IC). Overexpression of N1-IC led to a dose-dependent increase of NF-κB reporter activity (Figure 6A). Conversely, Notch1 silencing by siRNA significantly decreased endogenous NF-κB-driven luciferase activity (Figure 6B).

Next, we interrogated the role of Notch1 in c-IAP2 expression in TNBC cells. We had previously shown that Notch1 regulates NF-κB-induced c-IAP2 expression in cervical cancer cells in an IKKα-dependent fashion (35). Studies indicate that c-IAP2 is frequently overexpressed in cancers and contributes to tumorigenesis, chemoresistance and disease progression (65). Synthetic cIAP2 inhibitors cause massive apoptosis in MDA-MB-231 cells and complete remissions in MDA-MB-231-derived xenografts (66, 67). We studied cIAP2 expression upon activation of Notch signaling under the same conditions we used in other models, by co-culturing MDA-MB-231 cells with mouse fibroblasts engineered to express Notch ligand Jagged-1 (LTK-JAG) or control vector-transduced parental fibroblast (LTK-PAR) as we previously reported (37). Jagged1 exposure markedly induced cIAP-2 expression (Figure 6C). Knockdown of either Notch1 or IKKα suppressed this induction (Figure 6C). Notch4 knockdown had no effect, suggesting that not all Notch paralogs induce cIAP-2 in this system. Transfection efficiency was validated by Western blot using the same lysates used in luciferase experiments (Figures 6G–J).

We previously described that Notch1 and IKKα are recruited to the cIAP-2 promoter in cervical cancer cells (35). Similarly, co-culture of MDA-MB-231 cells with LTK-JAG fibroblasts induced prominent recruitment of Notch1, p50, p65, and IKKα to the cIAP-2 promoter compared to parental LTK fibroblasts (Figures 6D–F). Knockdown of Notch1 abrogated the recruitment p50, p65, and IKKα (Figures 6D–F). Taken together, these results suggest that Notch1 and IKKα cooperatively promote NF-κB transcriptional activity in MDA-MB-231 cells as they do in cervical cancer cells. Together with our data in luminal breast cancer cells (37) and the observations of the Espinosa group (36, 51), these observations suggest that IKKα mediates Notch1 nuclear effects in multiple cancer cell types, and may be a key, druggable mediator downstream of Notch in cancers where Notch1 is activated.

## Discussion

High expression of Notch1 and /or Jagged1 has been correlated with poor prognosis in TNBC (7). Dysregulation of canonical Notch signaling pathway has been also reported in many other tumors including breast cancer, and consequently, several structurally and pharmacologically distinct GSIs have been investigated in preclinical and clinical setting, particularly to target cancer stem cells (4). However, despite the recognized importance of Notch signaling in tumor biology, clinical evidence of efficacy with Notch-pathway inhibitors remains relatively limited. This is hardly uncommon for targeted agents, which tend be selectively effective only in tumors that rely on the targeted pathways. Precision medicine approaches based on mutational status and/or gene or protein expression profiling are necessary to identify tumors most likely to be sensitive to inhibition of specific pathways. Additionally, the phenotypic plasticity and clonal heterogeneity of many tumors make sustained single agent efficacy unlikely. Rational combination strategies based on mechanism are more likely to deliver efficacy at sub-toxic doses of targeted agents.

In the case of Notch inhibitors, only recently are clinical studies beginning to stratify tumors based on reliable biomarkers of Notch activity. This stratification must be tumor subtype-specific, as Notch signaling is highly context-dependent, and very few Notch signaling pathway targets are uniformly valid across tumor types. Additionally, the systemic toxicity of non-selective GSIs limits our ability to expose tumors to sustained and complete inhibition of Notch signaling. The identification of key, druggable pathway nodes upstream or downstream of Notch signaling in cancer cells or CSC could provide alternate strategies to therapeutically target this crucial pathway, or at least to design rational combinations that allow more selective targeting of the Notch pathway at lower doses of individual inhibitors. We and others have identified IKKα as a mediator of Notch oncogenic activities in a variety of malignancies, where IKKα mediates non-canonical, NF-κB-dependent gene expression (34–36, 68) as well as ERα-dependent gene expression (37). The data we describe here support a model whereby IKKα mediates the activation of AKT and mitochondrial metabolism as well as NF-κB-dependent transcription of anti-apoptotic genes in some TNBCs. Importantly, IKK pharmacological inhibition decreased AKT phosphorylation even in cells where GSI did not have this effect, suggesting that multiple pathways converge on the IKKα-AKT axis.

**Figure 7:**
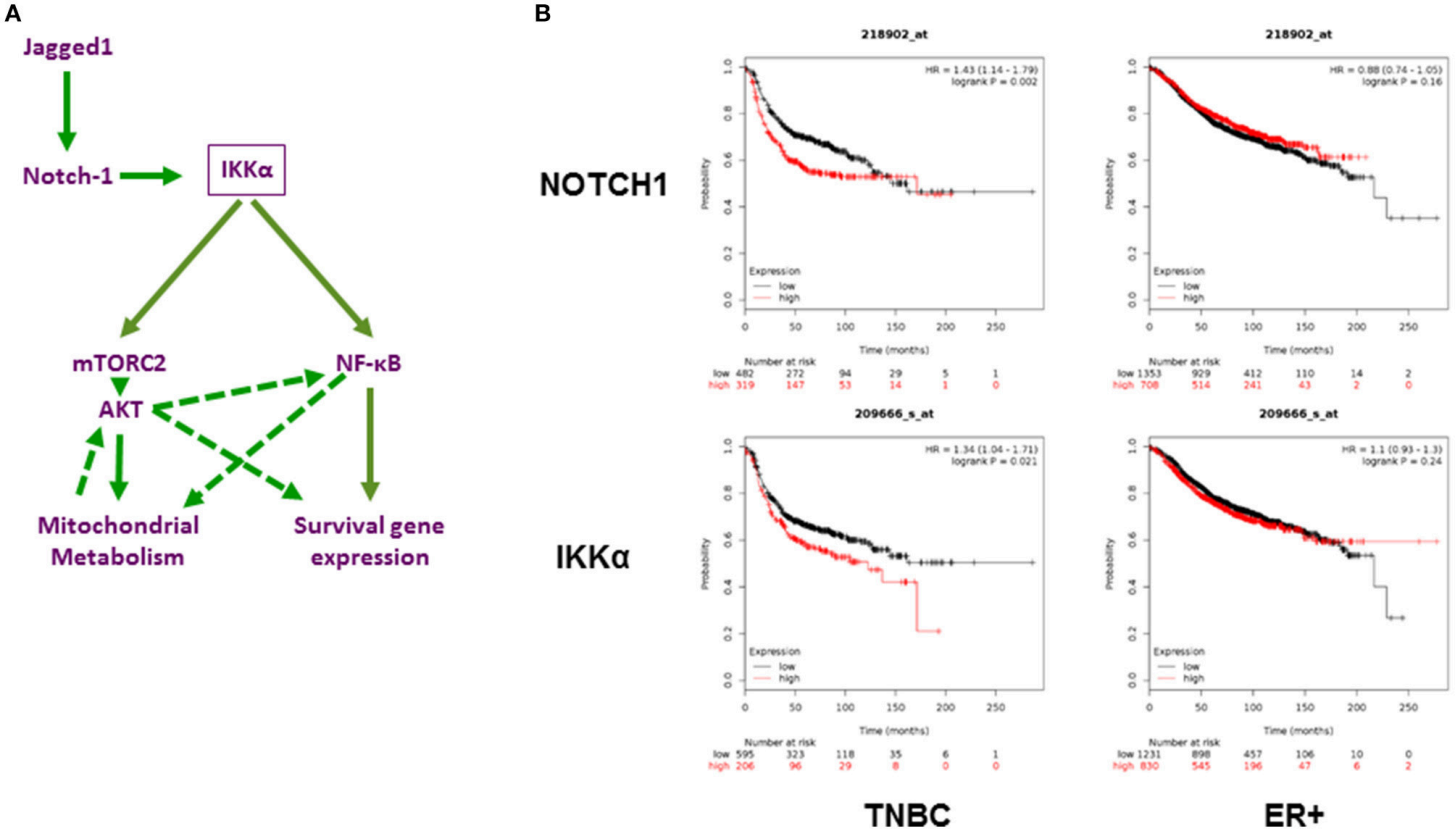
Role of Notch1 and IKKα in TNBC. **(A)** Working hypothesis: IKKα as a central mediator of mitochondrial and nuclear non-canonical Notch signaling in TNBC. Diagram representing our working hypothesis whereby Jagged1 activates Notch1, which in turn acts through IKKα to trigger two parallel and potentially interacting non-canonical pathways: a mitochondrial pathway culminating in mTORC2-dependent AKT activation and increased oxidative phosphorylation and a nuclear pathway whereby IKKα binds to NF-κB-responsive elements and triggers transcriptional activation of survival genes, such as c-IAP2. Dashed arrows indicate possible secondary effects of AKT on NF-κB activation and survival gene expression, which have been documented in the literature in other systems but not explored in this study. **(B)** Expression of NOTCH1 and IKKα (Gene symbol (CHUK) significantly correlates with poor relapse-free survival in TNBC but not ER^+^ breast cancers. Using the Kaplan-Meier Plotter (http://kmplot.com/analysis/; 73) Breast Cancer 2017 dataset, the correlation between Relapse Free Survival (RFS) and expression of these two transcripts was determined. TNBC (*n* = 801) and ER^+^ tumors (*n* = 2565) were analyzed separately.

The PI3K/AKT/mTOR pathway is one of the most actively investigated oncogenic pathways, and drugs that target this pathway are in various stages of clinical development (69), including in TNBC (70, 71). Recently, a phase 1 clinical study has evaluated targeting the PI3K/AKT/mTOR pathway using rapamycin in combination with liposomal doxorubicin and bevacizumab for the treatment of mesenchymal TNBC (72). Responses were limited to tumors with altered pathway activity. Importantly, resistance to PI3K/AKT/mTOR inhibitors in TNBC has been linked to Notch1 (6). Our findings suggest two possible, non-mutually exclusive mechanisms for such resistance, namely, activation of AKT by Notch1 via IKKα and mTORC2 and activation of NF-κB-dependent survival pathways. Figure 7A depicts our working model in diagram form. The AKT arm of this pathway is consistent with what has been described in glioma stem cells and Drosophila neuroblasts, whereby Notch1 associates with PTEN-induced kinase 1 (PINK1) on the mitochondrial surface to modulate mitochondrial function, and activates mTORC2/AKT signaling (31). In our model, this process requires IKKα, which activates mTORC2 by phosphorylating Rictor in a variety of other systems (52, 53). In turn, the activation of AKT modulates mitochondrial metabolism and oxidative phosphorylation in bulk TNBC cells as well as mammospheres. Contrary to their non-CSC counterparts, which rely largely on aerobic glycolysis for ATP production, breast CSC from multiple models including MDA-MB-231 have been reported to have higher levels of mitochondrial activity and ATP production from oxidative phosphorylation and less lactate production (60). This reliance on oxidative phosphorylation was attributed to the low proliferative activity of breast CSC. Our findings are consistent with those reported by Vlashi et al. (60). In our model, Notch1, IKKα, and AKT are required for enhanced oxidative phosphorylation in mammospheres. The metabolic substrate(s) used for Notch-induced oxidative phosphorylation in our system may include glucose but also glutamine. Of note, Notch1 induces glutaminolysis in T-ALL cells (74). Combined inhibition of Notch cleavage and IKK activity, or Notch cleavage and AKT activity results in virtual abolition of mammosphere growth in different models of TNBC, including a novel patient-derived line. Of note, this was also true in cells where GSI did not decrease pAKT, indicating that additional pathways downstream of Notch contribute to CSC survival. Our data indicate that one of these pathways is NF-κB.

Several studies have suggested that NF-κB is important in breast cancer initiation and progression (75–77). NF-κB promotes survival in numerous cancers cells, we and others have demonstrated that Notch activates it in numerous immune and neoplastic cells (35, 37, 78–80). Non-canonical Notch signaling has been shown to activate IL-6/JAK/STAT signaling in breast tumor cells in a NF-κB dependent manner (81). This pathway is considered a promising therapeutic target in TNBC (82). We previously reported that Notch1 promotes cIAP2 transcription by NF-κB, and associates with IKKα at NF-κB-dependent promoters in cervical cancer cell lines (35). The results we present herein indicate that this pathway is not exclusive to the models we originally studied, and has broader relevance to other cancers where Notch1 and NF-κB are active. Of note, there are possible positive feedback mechanisms that could reinforce these pathways. NF-κB can induce expression of Notch ligand Jagged1 (83, 84), and AKT can further activate IKKα (46, 85–87). Moreover, mitochondrial stress can activate AKT1 and NF-κB through mitochondrial retrograde signaling (88, 89). This effect may contribute to the increased AKT and NF-κB activity downstream of Notch1. Conversely, NF-κB can promote oxidative metabolism in breast cancer cells (90).

CSL-independent, non-canonical Notch signaling processes have been described in numerous systems, in neoplastic and non-neoplastic cells (31–33, 81, 91). Our work indicates that IKKα is a critical mediator of multiple non-canonical Notch signals in TNBC cells and TNBC CSC, controlling AKT activation, mitochondrial metabolism and NF-κB-dependent transcription. Consistent with our model, both Notch1 and IKKα (CHUK) mRNA expression are strongly associated with poor survival in TNBC but not ER^+^ breast cancers (Figure 7B).

We recently evaluated different GSIs for anti-mammosphere activity in TNBC models, and found that PF-3084014 was the most potent (62). Using two different TNBC cell lines, one PTEN-wild type and the other PTEN-null, we show here that combinations including a GSI and AKT inhibitor MK2206 or GSI and IKK inhibitor BAY-11-7082) potently inhibit mammosphere formation from sorted breast CSC and are more effective than any of the single agents tested.

Different markers have been used to identify breast CSC, including CD44^+^CD24^low^ (63), Aldehyde Dehydrogenase (ALDH1) (92), and as well as CD90^hi^ (64). Interestingly, using public domain gene expression profiles and Kaplan-Meier Plotter [http://kmplot.com/analysis/] we confirmed that CD90 expression is more significantly correlated with poor prognosis in ER^−^ breast cancer patients (Supplemental Figure 4), as described by Lu et al. (64). Using the original Lehmann TNBC subtype (11) classification, we find that CD90 expression correlates with poor prognosis in mesenchymal, mesenchymal-stem like and luminal androgen receptor subtypes but not in BL1, BL2, or immunomodulatory subtypes (Supplemental Figure 5). The recent refinement of TNBC molecular subtyping (12) has unified the M and MSL subtypes and eliminated the IM subtype, the latter resulting from immune cell infiltration of other subtypes. These data suggest that cancer stem-like cell markers may not be uniformly applicable to all TNBCs. The two mechanism-based combinations we explored in this study were highly effective in blocking secondary mammospheres formation from sorted CD90^hi^ or CD44^+^CD24^low^ cells, as well as in a patient-derived xenograft model.

Selective IKKα inhibitors are not yet available. However, numerous small molecule IKK inhibitors have been developed (93), and are a subject of active investigation (94), particularly as anti-inflammatory agents. Our data indicate that such agents would have potential as anti-CSC agents in TNBC. AKT inhibitors are currently in clinical development, and the agent we used in this study is in phase 2 clinical trials (95). Overall, our data indicate that AKT inhibitors and particularly IKK inhibitors would have potential therapeutic applications in combination with or as substitutes for Notch inhibitors, targeting critical effectors of Notch signaling in TNBC stem cells, and likely in other malignancies.

## Author Contributions

FH performed experiments, data analysis, and wrote complete draft of manuscript. CS performed experiments. DU performed experiments. YP performed experiments. DW guided flow cytometry and performed flow sorting experiments. JC provided expertise in mammosphere assays. JZ supervised quantitative ChIP experiments. SM supervised CS. LD developed protocols for, performed and interpreted confocal microscopy experiments. MM developed PDX model, performed experiments with it. MB developed PDX model. BC-B led PDX development protocol. AP analyzed data, supervised YP. LMM contributed to study design and data analysis, reviewed manuscript. TG contributed to GSI experiments design and analysis, reviewed manuscript. BO contributed to study design and analysis, reviewed manuscript. LM developed overall experimental plan, supervised experiments and data analysis, reviewed and edited manuscript.

### Conflict of Interest Statement

The authors declare that the research was conducted in the absence of any commercial or financial relationships that could be construed as a potential conflict of interest.

## Supplementary Material

Supplemental Figure 1 Co-culture of MDA-MB-231 cells with LTK-JAG or Notch1 activation with EDTA induces AKT and IKKα phosphorylation. **(A)** MDA-MB-231 cells were co-cultured with mouse fibroblast parental LTK cells (PAR) (P) or LTK cells overexpressing Jagged-1 (JAG) (J) for the indicated times. Whole cell lysates were analyzed by Western blotting. **(B)** Following co-culture as described above, 1000 μg of cell lysate were immunoprecipitated with a human-specific IKKα antibody. PAR cell lysates were immunoprecipitated as a negative control. S180 P-IKKα and human-specific IKKα were immunoblotted following immunoprecipitation. **(C)** MDA-MB-231 cells were transfected with scrambled siRNA or Notch1 siRNA as described in Figure 7. Forty-eight hours after transfection, cells were treated with 5 mM EDTA for the indicated times. Cleaved Notch1, S473 pAKT, total AKT, pIKKα/β, total IKKα and actin were analyzed by Western blotting.

Supplemental Figure 2 Jagged1 induces S476 AKT phosphorylation in some but not all TNBC lines. BT-549 (Mesenchymal), MDA-MB-453 (Luminal Androgen Receptor, LAR) and HCC1806 (Basal-Like 2) cells were plated on 0.2% gelatin (Control) or human recombinant 1μg/ml Jagged1 in gelatin (Jagged)-coated plates in the presence of the indicated drugs: AKT inhibitor MK-2206 (5 μM), GSI PF-03084014 (5 μM), IKK inhibitor BAY11-7082 (5 μM), mTORC1 selective inhibitor Everolimus (5 μM), and dual mTORC1/mTORC2 inhibitor KU-0063794 (5 μM) for an hour. Whole cell lysates were analyzed by Western blotting.

Supplemental Figure 3 MDA-MB-231 cellular metabolism is dependent on Notch1 and IKKα in basal condition. MDA-MB-231 cells were transfected with control siRNA, Notch1siRNA or IKKα siRNA. Forty-eight hours following transfection, equal numbers of live cells were plated on a Control XF24 cell culture plate (0.2% gelatin) and analyzed for OCR and ECAR by Seahorse Analyzer as described in the Methods section.

Supplemental Figure 4 Cancer Stem-like cells marker CD90 significantly predicts poor survival in TNBC. Using the Kaplan-Meier Plotter Breast Cancer 2017 dataset, Relapse Free Survival (RFS) of TNBC (*n* = 801) was determined. CD90 gene symbol (213869_x_at) was used to determine RFS in ER positive and TNBC subtypes using the median value to dichotomize patients.

Supplemental Figure 5 CD90 predicts poor survival in some but not all TNBC molecular subtypes. Using the Kaplan-Meier Plotter Breast Cancer 2017 dataset and the original 7 Lehmann-Pietenpol subtypes (*n* = 1246), the correlation between Relapse Free Survival (RFS) and CD90 expression was determined. Basal-Like 1 (BL-1), Basal-Like 2 (BL-2), Immunomodulatory (IM), Mesenchymal (M), Mesenchymal Stem-like (MSL) and Luminal Androgen receptor (LAR) TNBC subtypes are shown separately.

Supplemental Figure 6 GSI (PF-03084014) in combination with an AKT inhibitor or an IKK inhibitor is effective against PDX-derived mammospheres. **(A)** Baseline expression of Jagged1, Notch1, Notch3 and Hey1 in PDX derived cell line (2K1) was measured by RT-PCR. **(B)** PDX Mammospheres were enriched from 2K1 cells as described earlier, and P1 PDX mammospheres were treated with GSI PF-03084014 (PF, 5 μM) or AKT inhibitor MK-2206 (MK, 5 μM) or IKK inhibitor Bay11-7082 (Bay11, 1μM) as single agents or with combinations including PF (5 μM) plus MK (5 μM), or PF (5 μM) plus Bay11 (1 μM) for one week (twice per week treatment). Following incubation mammospheres were counted using a Nikon microscope.
