# Supplementary figures and images for: Notch Signaling Regulates Mitochondrial Metabolism and NF-κB Activity in Triple-Negative Breast Cancer Cells via IKKα-Dependent Non-canonical Pathways
Source: Front Oncol. 2018 Dec 4;8:575. doi: 10.3389/fonc.2018.00575 (PMC6289043; doi:10.3389/fonc.2018.00575)

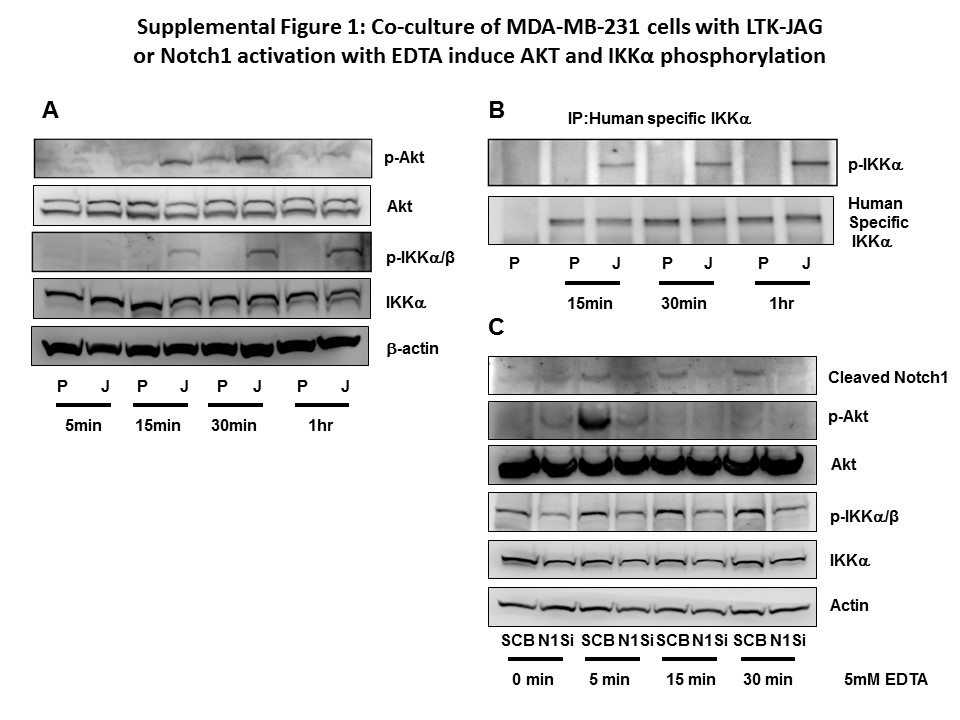

Supplement: Supplemental Figure 1 — Co-culture of MDA-MB-231 cells with LTK-JAG or Notch1 activation with EDTA induces AKT and IKKα phosphorylation. (A) MDA-MB-231 cells were co-cultured with mouse fibroblast parental LTK cells (PAR) (P) or LTK cells overexpressing Jagged-1 (JAG) (J) for the indicated times. Whole cell lysates were analyzed by Western blotting. (B) Following co-culture as described above, 1000 μg of cell lysate were immunoprecipitated with a human-specific IKKα antibody. PAR cell lysates were immunoprecipitated as a negative control. S180 P-IKKα and human-specific IKKα were immunoblotted following immunoprecipitation. (C) MDA-MB-231 cells were transfected with scrambled siRNA or Notch1 siRNA as described in Figure 7. Forty-eight hours after transfection, cells were treated with 5 mM EDTA for the indicated times. Cleaved Notch1, S473 pAKT, total AKT, pIKKα/β, total IKKα and actin were analyzed by Western blotting. [file Image_1.JPEG]

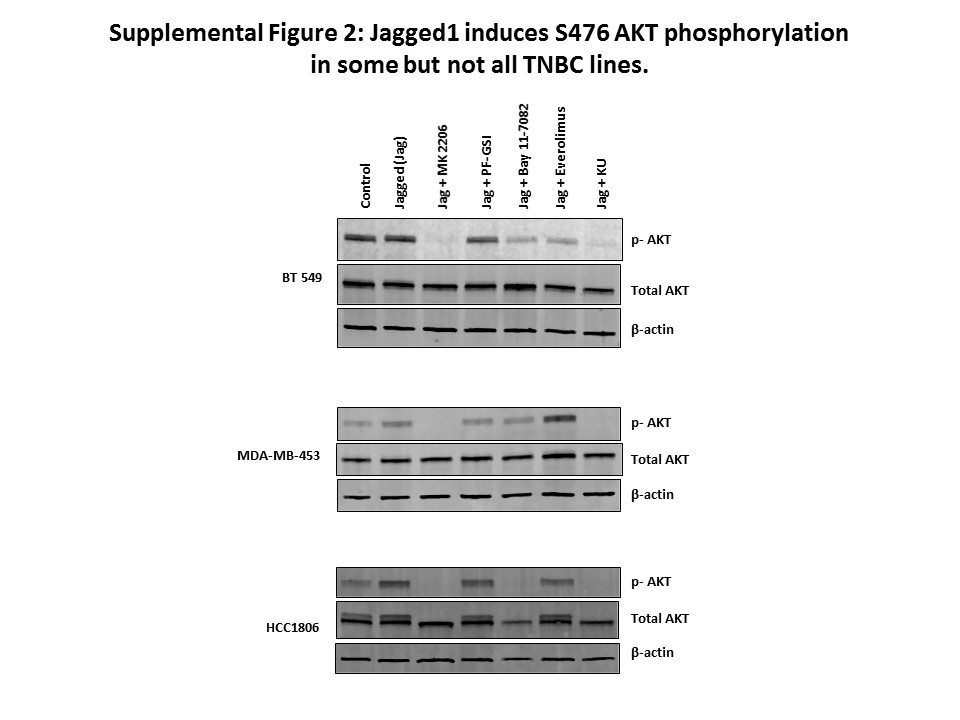

Supplement: Supplemental Figure 2 — Jagged1 induces S476 AKT phosphorylation in some but not all TNBC lines. BT-549 (Mesenchymal), MDA-MB-453 (Luminal Androgen Receptor, LAR) and HCC1806 (Basal-Like 2) cells were plated on 0.2% gelatin (Control) or human recombinant 1μg/ml Jagged1 in gelatin (Jagged)-coated plates in the presence of the indicated drugs: AKT inhibitor MK-2206 (5 μM), GSI PF-03084014 (5 μM), IKK inhibitor BAY11-7082 (5 μM), mTORC1 selective inhibitor Everolimus (5 μM), and dual mTORC1/mTORC2 inhibitor KU-0063794 (5 μM) for an hour. Whole cell lysates were analyzed by Western blotting. [file Image_2.JPEG]

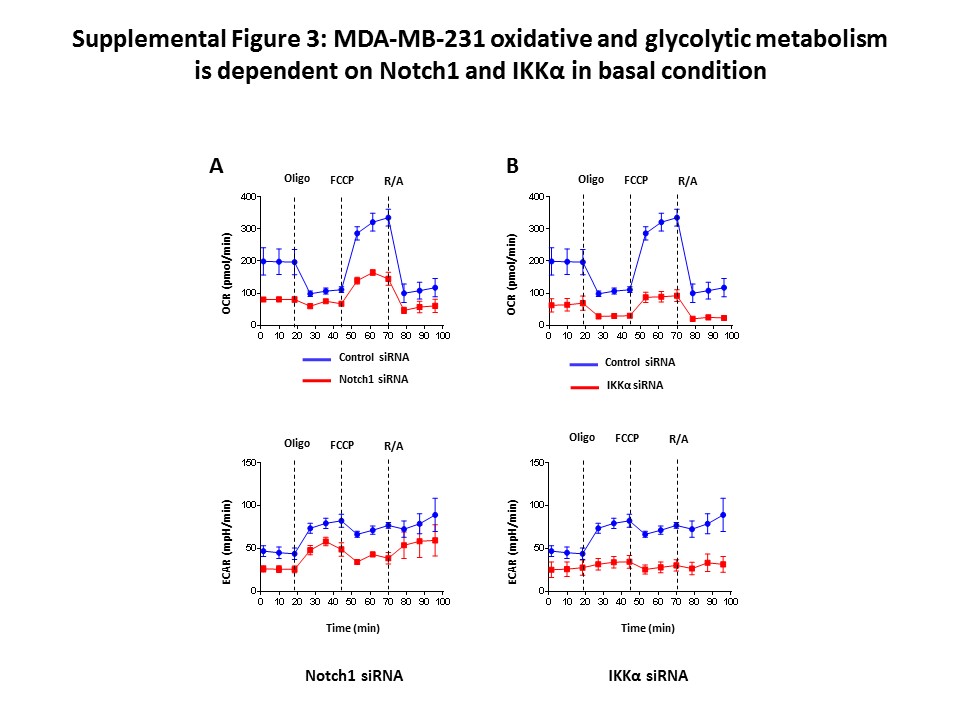

Supplement: Supplemental Figure 3 — MDA-MB-231 cellular metabolism is dependent on Notch1 and IKKα in basal condition. MDA-MB-231 cells were transfected with control siRNA, Notch1siRNA or IKKα siRNA. Forty-eight hours following transfection, equal numbers of live cells were plated on a Control XF24 cell culture plate (0.2% gelatin) and analyzed for OCR and ECAR by Seahorse Analyzer as described in the Methods section. [file Image_3.JPEG]

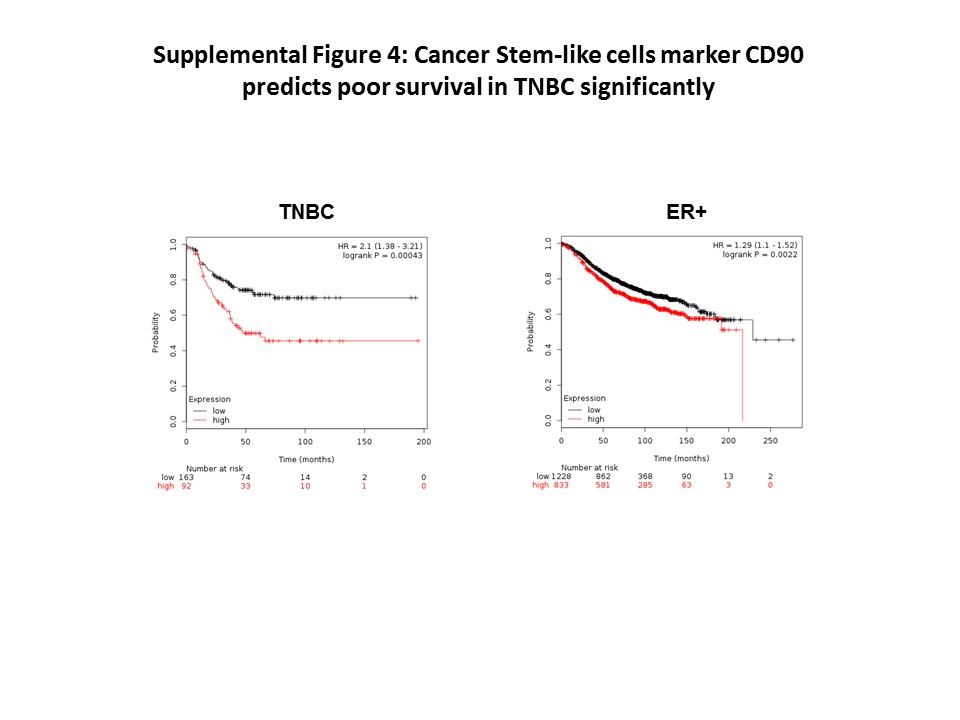

Supplement: Supplemental Figure 4 — Cancer Stem-like cells marker CD90 significantly predicts poor survival in TNBC. Using the Kaplan-Meier Plotter Breast Cancer 2017 dataset, Relapse Free Survival (RFS) of TNBC (n = 801) was determined. CD90 gene symbol (213869_x_at) was used to determine RFS in ER positive and TNBC subtypes using the median value to dichotomize patients. [file Image_4.JPEG]

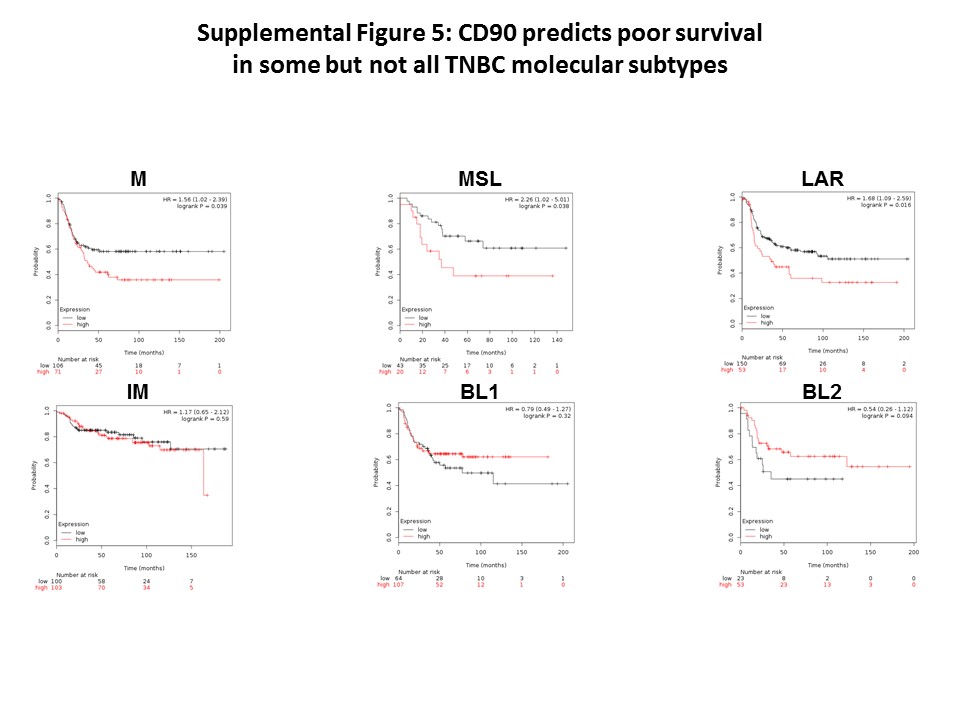

Supplement: Supplemental Figure 5 — CD90 predicts poor survival in some but not all TNBC molecular subtypes. Using the Kaplan-Meier Plotter Breast Cancer 2017 dataset and the original 7 Lehmann-Pietenpol subtypes (n = 1246), the correlation between Relapse Free Survival (RFS) and CD90 expression was determined. Basal-Like 1 (BL-1), Basal-Like 2 (BL-2), Immunomodulatory (IM), Mesenchymal (M), Mesenchymal Stem-like (MSL) and Luminal Androgen receptor (LAR) TNBC subtypes are shown separately. [file Image_5.JPEG]

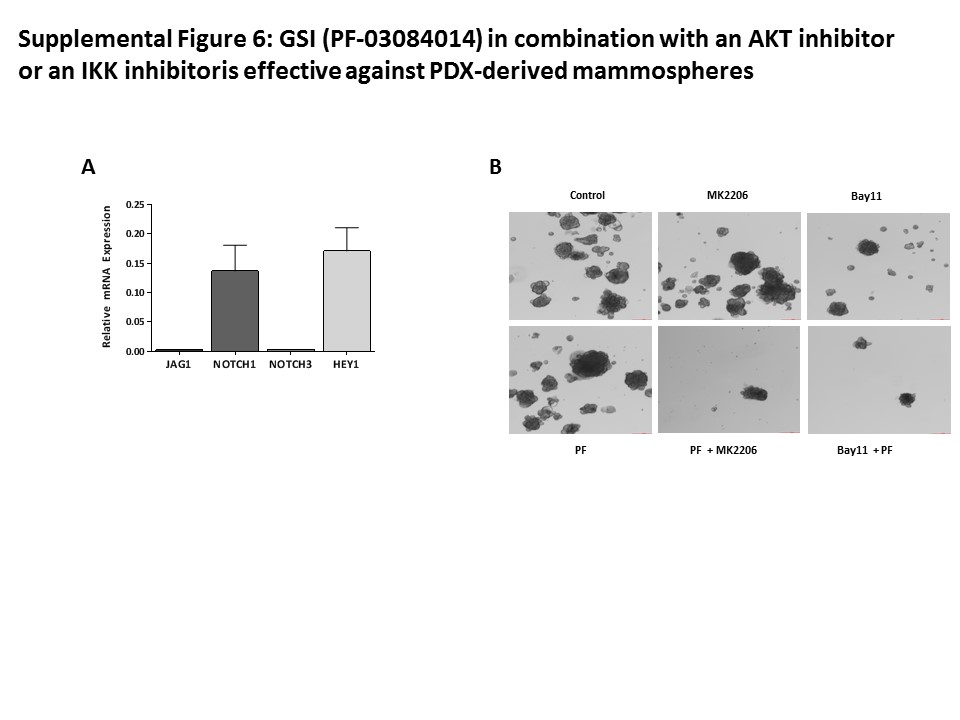

Supplement: Supplemental Figure 6 — GSI (PF-03084014) in combination with an AKT inhibitor or an IKK inhibitor is effective against PDX-derived mammospheres. (A) Baseline expression of Jagged1, Notch1, Notch3 and Hey1 in PDX derived cell line (2K1) was measured by RT-PCR. (B) PDX Mammospheres were enriched from 2K1 cells as described earlier, and P1 PDX mammospheres were treated with GSI PF-03084014 (PF, 5 μM) or AKT inhibitor MK-2206 (MK, 5 μM) or IKK inhibitor Bay11-7082 (Bay11, 1μM) as single agents or with combinations including PF (5 μM) plus MK (5 μM), or PF (5 μM) plus Bay11 (1 μM) for one week (twice per week treatment). Following incubation mammospheres were counted using a Nikon microscope. [file Image_6.JPEG]
